# Direct Evidence for the Feedforward Neurovascular Coupling Mechanism in Humans During Task Onset: An EEG-fNIRS-TCD Multimodal Imaging Study

**DOI:** 10.3390/s26061790

**Published:** 2026-03-12

**Authors:** Joel S. Burma, Matthew G. Neill, Elizabeth K. S. Fletcher, Jina Seok, Nathan E. Johnson, Kathryn J. Schneider, Chantel T. Debert, Jeff F. Dunn, Jonathan D. Smirl

**Affiliations:** 1 Cerebrovascular Concussion Laboratory, Faculty of Kinesiology, University of Calgary, Calgary, AB T2N 1N4, Canadajina.seok@ucalgary.ca (J.S.); jonathan.smirl@ucalgary.ca (J.D.S.); 2 Libin Cardiovascular Institute of Alberta, University of Calgary, Calgary, AB T2N 1N4, Canada; 3 Alberta Children’s Hospital Research Institute, University of Calgary, Calgary, AB T2N 1N4, Canada; 4 Hotchkiss Brain Institute, University of Calgary, Calgary, AB T2N 1N4, Canada; 5 Integrated Concussion Research Program, University of Calgary, Calgary, AB T2N 1N4, Canada; 6 Sport Injury Prevention Research Centre, Faculty of Kinesiology, University of Calgary, Calgary, AB T2N 1N4, Canada; 7 Human Performance Laboratory, Faculty of Kinesiology, University of Calgary, Calgary, AB T2N 1N4, Canada; 8 Faculty of Rehabilitation Medicine, Department of Physical Therapy, University of Alberta, Edmonton, AB T6G 2R3, Canada; 9 Department of Clinical Neurosciences, Cumming School of Medicine, University of Calgary, Calgary, AB T2N 1N4, Canada; 10 Department of Radiology, Cumming School of Medicine, University of Calgary, Calgary, AB T2N 1N4, Canada

**Keywords:** neurovascular coupling, sex differences, electroencephalography, functional near-infrared spectroscopy, transcranial Doppler ultrasound

## Abstract

**Highlights:**

**What are the main findings?**

- Concurrent EEG, fNIRS, and TCD measurements showed that neuronal activity and microvascular oxygenation predicted upstream arterial blood flow during task onset, supporting a unidirectional feedforward neurovascular coupling response.
- The overall NVC response was consistent across participants, with minimal influence of concussion history, mental health, or learning disabilities, although sex, age, and cardiorespiratory fitness influenced baseline and peak hemodynamic values.

**What is the implication of the main findings?**

- This study represents the first successful concurrent assessment of neuronal, microvascular, and intracranial arterial responses to quantify neurovascular coupling in humans; providing support for the feedforward neurovascular coupling model in which neuronal activation and microvascular changes drive upstream cerebrovascular responses during cognitive and motor tasks.
- The findings demonstrate the utility of multimodal imaging (EEG–fNIRS–TCD) for characterizing integrated neurovascular responses in humans, providing a framework for future studies of cerebrovascular and neurological health.

**Abstract:**

This investigation assessed the neurovascular coupling response through integrated assessments of neuronal function [electroencephalography (EEG)], microvascular oxygenation concentrations [functional near-infrared spectroscopy (fNIRS)], and arterial responses [transcranial Doppler ultrasound (TCD)]. The NVC response was assessed in 113 participants (86 females, aged 19–40 years) during visual (“Where’s Waldo?”) and motor (finger tapping) tasks. Block-averaged, time–frequency power was computed from the EEG data, while hemodynamic response functions were obtained from the fNIRS and TCD metrics. Granger causality assessed the predictiveness between EEG-fNIRS-TCD waveforms for each participant and was converted into a percentage of individuals displaying a significant value. Linear models were computed to determine the influence of sex, concussion history, young adulthood age, cardiorespiratory fitness, and mental health/learning disabilities on NVC parameters. During the initial 10 s of task onset, unidirectional predictiveness was weak to very strong for EEG-TCD (range: 47–83%) and fNIRS-TCD (44–92%) relationships; however, very weak to weak predictiveness was seen for the E0EG-fNIRS (0–29%) relationship for both tasks. Aside from known sex-, age-, and fitness-based influences on baseline/peak hemodynamic values (*p* < 0.050), the addition of concussion history and mental health/learning disabilities had minimal influence on NVC responses (*p* > 0.050). The findings demonstrated a unidirectional feedforward mechanism from the neuronal and microvasculature to the upstream arteries during task onset.

## 1. Introduction

Neurovascular coupling (NVC), also known as functional hyperemia, describes the tightly regulated relationship between neuronal activity and cerebral perfusion, which ensures that active regions of the brain parenchyma receive sufficient oxygen and nutrients to meet metabolic demands during activation [1,2,3]. This response is critical for maintaining optimal brain function and relies on feedforward and feedback mechanisms [4]. The feedforward component is characterized by rapid adjustments in blood delivery to activated regions, while feedback mechanisms maintain cerebral homeostasis by modulating vascular tone in response to metabolic byproducts [4]. Previous work from the author group has demonstrated the feasibility of multimodal electroencephalography (EEG), functional near-infrared spectroscopy (fNIRS), and transcranial Doppler ultrasound (TCD) to assess the NVC response to motor- and visual-based tasks in a small pilot study (*n* = 15) [5]. However, a large-scale investigation characterizing NVC using concurrent assessments of neural and hemodynamic systems in humans has not been completed due to the difficulty of simultaneous integration.

Several investigations have noted that neuronal or hemodynamic variables are influenced by modifying variables such as biological sex, age, concussion history, and cardiorespiratory fitness [6,7,8,9]. For example, while females have smaller brains, they contain more gray matter density due to greater cortical folding of the brain parenchyma [6,7]. Functional differences have additionally been noted, with females displaying larger EEG delta, theta, alpha, and beta power amplitudes [10,11]. Moreover, long-term neuronal deficits and hemodynamic alterations have been noted in those with a history of concussion; however, it is unclear how these influence the NVC response [12,13,14]. Linear declines in cerebral perfusion and neuronal density are known to occur with increasing age starting in young adulthood, with some investigations denoting differences in NVC metrics in older populations [15,16]. However, regular physical activity and exercise have been shown to have protective effects, attenuating the rate of neuronal/perfusion decline [17,18,19,20]. Finally, it is currently unknown how mental health and learning disabilities influence the NVC response.

Many investigations into these modifying variables have been conducted using unimodal neuroimaging techniques (isolated TCD, fNIRS, or magnetic resonance imaging), which only assess a singular aspect of cerebral functioning [21]. Therefore, the purpose of this study was to assess the directional relationship between neuronal function (bioelectric activity assessed via EEG), microvascular oxygenation changes (via fNIRS), and arterial changes (via TCD) during visual- and motor-based NVC in young adults. A secondary aim of this study is to assess the influence of sex, age, concussion history, and aerobic capacity on the NVC response. This comprehensive approach will enable an in-depth bidirectional assessment of the known feedforward and feedback loops in young adults. We hypothesize that multimodal assessment will provide objective concurrent evidence of feedforward NVC. Moreover, we hypothesize that NVC will be weaker in those with a history of concussion [8], older age [22], and lower aerobic capacity [23].

## 2. Materials and Methods

### 2.1. Ethics and Study Design

The University of Calgary Conjoint Health Research Ethics Review Board approved the study under protocol REB20-2112. Except for study registration within a public database, all procedures adhered to the principles outlined in the 2013 revision of the Declaration of Helsinki [24]. A convenience sample of healthy young adult volunteers was recruited using word of mouth and snowball sampling techniques. Data collection followed the guidelines and standards recommended in the Neurovascular Coupling White Paper and other recommendations [3,21,25]. A detailed description of all methods and the full study design flowchart can be found in the previously published protocol paper [26].

### 2.2. Participants

The participants completed a main multimodal neuroimaging visit (*n* = 113; 77% female; 23 ± 5 years, range: 19–40 years; body mass index: 24 ± 3 kg/m^2^) and a subsample additionally completed an optional maximal oxygen uptake test (VO_2max_) (*n* = 58; 77% female; 23 ± 6 years, range: 19–40 years; body mass index: 24 ± 3 kg/m^2^) (Table 1). The exercise test was completed within a median of 2 days (interquartile range: 1–3, range: 0–10) of the multimodal imaging visit. Testing for female participants occurred between days 3 and 10 of the early follicular phase, during which 11 used oral contraceptives, 33 used intrauterine devices, and 42 did not use hormonal contraceptive methods. All participants were free from chronic neurological, cerebrovascular, cardiovascular, and respiratory conditions.

Data collection occurred between 07:00 and 18:00 from March 2023 to May 2024 in a controlled environment: barometric pressure (666 ± 6 Torr), temperature (22 ± 1 °C), and humidity (22 ± 12 percent). For 12 h prior to data collection, the participants abstained from nicotine products, caffeine, cannabis, alcohol, and other illicit substances. The participants also avoided exercise for at least 6 h prior to each visit [27]. To control for ambient light impacting the fNIRS signals, testing was completed in a darkened room (lumens range: 30–100, equivalent to 2–8 candles; LUX LED Light Meter LM-50KL, LATNEX, Woodbridge, ON, Canada).

### 2.3. Instrumentation

**Figure 1:**
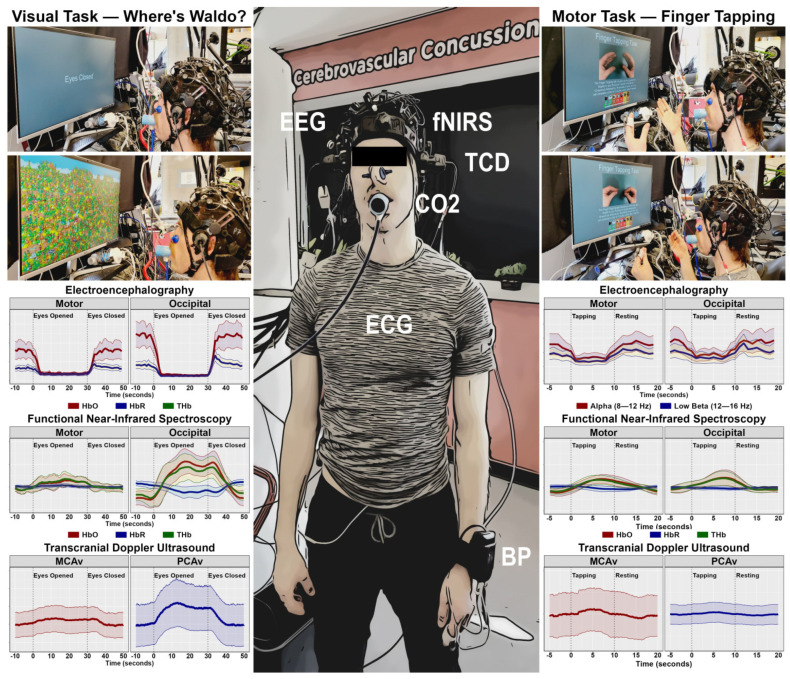
A representative photo of a participant attached to all neuroimaging and physiological equipment, demonstrating the visual (**left panels**) and motor (**right panels**) tasks and their associated time–frequency and hemodynamic responses in the motor and occipital regions. Electroencephalography (EEG), functional near-infrared spectroscopy (fNIRS), transcranial Doppler ultrasound (TCD), carbon dioxide (CO_2_), electrocardiography (ECG), blood pressure (BP), oxygenated hemoglobin (HbO), deoxygenated hemoglobin (HbR), and total hemoglobin (THb). Figure reproduced with permission [3,5].

An in-depth description of the multimodal imaging modality, along with a visualization of the montage, has been published elsewhere [26]. EEG electrodes and fNIRS optodes were held in place using a custom-designed headcap, with Cz positioned over the vertex of the head (i.e., the midpoint between preauricular points and the naison/inion) (Figure 1). EEG data were collected at 1000 Hz using a 16-channel Cyton Biosensing Board (OpenBCI, Brooklyn, NY, USA) and sent to an OpenBCI GUI. Ground and reference electrodes were positioned over the left and right mastoid processes, respectively [28]. A continuous-wave NIRS device (cwNIRS) was used to sample fNIRS data at 3.91 Hz from 16 sources and 16 detectors (NIRScout; NIRx Medical Technologies, Berlin, Germany), which created 40 channels (20 per hemisphere) and were collected using the NIRStar GUI (Figure 1) [29,30]. Two 2 MHz TCD ultrasound probes were held in place with an adjustable headframe (DWL Technologies, San Juan Capistrano, CA, USA) and positioned over the transtemporal windows (Figure 1) [29]. These probes insonated the middle cerebral and posterior cerebral arteries, which were confirmed using carotid compressions and simple visual tasks [31]. Beat-to-beat blood pressure was captured using a Finometer NOVA device (Finapres Medical Systems, Amsterdam, The Netherlands) and corrected using a height calibration unit (Finapres Medical Systems, Amsterdam, The Netherlands) [32,33]. Each PQRST waveform was captured using a 3-lead electrocardiography system, using lead II methodology (FE 201; AD Instruments, Colorado Springs, CO, USA). Breath-by-breath respiratory parameters were captured using an inline gas analyzer (ML206; AD Instruments, Colorado Springs, CO, USA). Physiological and TCD data were time-synchronized using the LabChart GUI (AD Instruments, Colorado Springs, CO, USA), which linearly interpolated the TCD (100 Hz) and Finapres NOVA (200 Hz) devices to 1000 Hz.

### 2.4. Experimental Protocols

This investigation was a subsection of a larger study additionally using the multimodal approach and the aforementioned equipment to assess dynamic cerebral autoregulation (squat-stand maneuvers), cerebrovascular reactivity (dynamic end-tidal forcing), and cardiovascular function (quiet sitting and standing) [26]. The current investigation included a motor finger-tapping task and a visual complex scene search (i.e., “Where’s Waldo/Wally?”). Pilot work has demonstrated the feasibility of the EEG-fNIRS-TCD approach to capture the NVC response using these tasks [5]. For the finger-tapping task, the participants completed 12 blocks consisting of 15 s of rest followed by 10 s of tapping their thumbs to their four fingers on both hands at a rate of 1 Hz (32–34). For the Waldo task, the participants completed 10 blocks of ~20 s with eyes closed and 40 s of active engagement with eyes open during the task [34,35]. Prior to completion, the participants were instructed to search for the five characters within the Waldo Universe (i.e., Waldo, Odlaw, Wenda, Wizard Whitebeard, and Woof’s tail), where a new puzzle was presented for each cycle (i.e., a total of 10) [36]. These tasks were employed to maximize engagement.

The fNIRS task instructions were automated to present the rest and finger tapping sections via PsychoPy [37]. With the Waldo task, the researchers manually input the triggers via PsychoPy, as the participants had their eyes closed and thus could not see visual cues. Additionally, this enabled the researchers to extend the resting portion in the presence of Mayer waves [38]. The PsychoPy platform also allowed for precise time synchronization across the three streaming platforms (OpenBCI GUI, NIRStar, and TCD) using a C-pod and a parallel port replicator. The methods paper for this project explicitly details the time synchronization process via PsychoPy, which allowed for the combination of datasets during offline analysis [26].

The participants completed a graded ramp exercise test on a cycle ergometer to quantify VO_2max_ [39,40]. The protocol included a 5-min warm-up at a fixed workload (40 W for females and 50 W for males), followed by a ramp protocol aimed to be 8–12 min in length [39,40]. For females, the workload increased by 15 W per minute; for males, it increased by 25 W per minute until volitional exhaustion. Standardized verbal encouragement was provided to ensure maximal effort.

### 2.5. Data Processing

The EEGLAB toolbox in MATLAB (version 2024.0) was used to filter, clean, and remove artifacts from the EEG data [41]. Specifically, a bandpass filter of 0.5 to 40 Hz was applied to reduce noise, particularly noise arising from nearby power lines (~60 Hz) and other neuroimaging and biomedical equipment. Artifact Subspace Reconstruction and Independent Component Analysis were subsequently used to identify, isolate, and correct artifacts arising from eye blinks, electrode movement, and muscle activity [41,42]. The Homer3 (version 1.80.2) software package was used to convert, clean, and remove motion artifacts from the fNIRS data [43]. The raw light voltages collected from the NIRScout were converted into optical densities and then transformed into chromophore concentrations (i.e., HbO, HbR, and THb) via the application of the modified Beer–Lambert law [43]. Signal quality was assessed using the Pollonini method, which evaluates the presence of cardiac pulsatility within each channel as an indicator of physiological signal integrity [44]. Compared to the scalp coupling index, which quantifies optode-scalp mechanical coupling based on wavelength correlation, the Pollonini approach verifies the presence of physiologically meaningful hemodynamic pulse oscillations [44]. Given the study’s focus on NVC and temporal dynamics, prioritizing physiological signal detection ensured that channels containing valid vascular information were retained for analysis. Using a cardiac pulsation cutoff of 0.6 Hz and a 2-s window, channels below this threshold were retained if a clear cardiac signal was present, as verified through frequency spectra and wavelet transform plots. This maximized the number of channels available for HRF analysis while maintaining data quality. Wavelet motion correction was then applied to identify and remove artifacts arising from head and/or optode movement [45]. Mean middle cerebral artery velocity (MCAv), posterior cerebral artery velocity (PCAv), and arterial pressure were determined by averaging data points from each individual pulsatile waveform [46]. From these traces, systolic values were determined from the peak of the pulsatile waveforms, while diastolic values were taken from the end-diastolic point of the waveform (i.e., the lowest value before the next upstroke). Partial pressure end-tidal carbon dioxisde (P_ET_CO_2_) was calculated as the peak expired value recorded during each breath. Heart rate was derived from the R-R intervals of the ECG, converted from milliseconds to beats per minute.

Oxygen uptake was measured breath by breath using a metabolic cart that analyzed the expired gas from the sampling chamber. The system continuously recorded minute ventilation, oxygen consumption, and carbon dioxide production. These variables were calculated based on the measured volumes of expired air and the concentrations of oxygen and carbon dioxide in the expired gas. The average respiratory exchange ratio (i.e., carbon dioxide expulsion to oxygen uptake) at the end of the exertion test was >1.10. Prior to each test, the metabolic cart was calibrated to ensure accuracy in flow rates and gas analysis. Oxygen uptake data were averaged over 15-s intervals to determine VO_2max_ throughout the exercise protocol [47,48].

### 2.6. Independent and Outcome Variables

The independent variables in the current investigation were sex, concussion history, relative VO_2_, age, and mental health and learning disability diagnoses. Diagnosed concussion history and the presence of a mental health or learning disability diagnosis were ascertained through reporting on the Athlete Background and Symptom Evaluation portions of the Sport Concussion Assessment Tool 6 [49,50,51]. Block averages were produced across all EEG, fNIRS, and TCD data, time-synchronized to task onset and averaged across all task trials. For finger tapping, neurodynamic and hemodynamic responses were produced 10 s before and after task onset, while for Waldo these responses were produced 10 s before and 30 s after task onset. The outcome variables were the NVC metrics obtained from the EEG-fNIRS-TCD multimodal approach, including baseline, peak, relative increases/decreases, absolute increases/decreases, area under the curve during task engagement (AUC).

Block averages were produced across all EEG, fNIRS, and TCD data, time-synchronized to task onset and averaged across all task trials. The block averages for EEG data were computed within the alpha (8–12 Hz) and beta 1 (12–16 Hz) frequency bands [52,53], as pilot work demonstrated that these frequency bands had the largest changes associated with these tasks [5]. Moreover, given the regions of interest, these were averaged across central electrodes (C3, Cz, and C4) for the finger-tapping task and occipital electrodes (PO3, PO4, O1, Oz, and O2). Concentrating on two frequency bands and combining data across electrodes within neural regions reduced the number of comparisons and maximized statistical power.

For the fNIRS data, hemodynamic response functions were calculated for all chromophores (HbO, HbR, and THb) in the channels that met the signal quality criteria. Previous research has shown that averaging fNIRS metrics across a region can reduce the sensitivity of detecting subtle physiological differences between groups or populations [54]. To address this, a single channel within the five regions was chosen for further analysis, and identified as the channel with the largest HbO AUC for each task. This maximized the signal-to-noise ratio, preserving the ability to compare physiological responses across EEG, fNIRS, and TCD data. Similar to the neural responses, to maximize statistical power, only the motor fNIRS and MCAv data were included in the finger tapping analysis and the occipital fNIRS and PCAv were included in the Waldo analysis. Pilot work has shown these regions are closely associated with the respective neural and hemodynamic demands of these tasks.

To enable comparison across modalities with inherently different sampling frequencies, all signals were time-locked to task onset and analyzed within standardized temporal windows rather than at their native sampling rates. Block-averaged responses were computed for each modality, and temporal metrics (baseline, peak, and AUC) were derived from these synchronized time series. By analyzing task-evoked changes within predefined windows rather than millisecond-level fluctuations, cross-device comparisons were performed on physiologically meaningful response epochs that reflect the slower vascular dynamics relative to bioelectrical activity. For EEG, fNIRS, and TCD data, baseline values were calculated as the average signal over the 10 s preceding task onset. Peak values were determined as the maximum signal within 10 s after task onset for the finger-tapping task and 30 s for the Waldo task. For EEG and TCD, both relative (percent change from baseline to peak) and absolute (difference between baseline and peak) increases or decreases were calculated, as these metrics are in absolute units. For fNIRS, only absolute changes were used, as the recorded units are relative concentrations. The AUC was computed as the cumulative area of the signal above baseline during the task engagement period—10 s for finger tapping and 30 s for the Waldo task [54]. EEG signals were analyzed within physiologically relevant frequency bands, including delta (0.5–4 Hz), theta (4–8 Hz), alpha (8–12 Hz), low beta (12–16 Hz), medium beta (16–20 Hz), high beta (20–30 Hz), and gamma (30–40 Hz). The EEG data were processed to align with the fNIRS timing by computing time–frequency power band analysis and averaging across 256 data points. The latter was also completed for the cerebral blood velocity (CBv) traces [46], ensuring that the temporal resolution of EEG and TCD data was comparable to fNIRS data while preserving critical timing information.

### 2.7. Sample Size Calculation

An a priori power analysis was conducted for a linear model with five predictor variables: sex, concussion history, age, relative VO_2max_, and mental health/learning disability composite [55]. Assuming a moderate effect size (*f*^2^ = 0.15), an alpha level of 0.05, and 95% power, the analysis indicated that a minimum of 89 participants would be required to detect significant effects. A second power analysis was conducted to determine that 98 participants would be required to detect a difference with a small effect size (*f*^2^ = 0.02), an alpha level of 0.05, and a desired statistical power of 80% [56]. Two power analyses were completed; for physiological findings, this ensures that both robust, easily detectable moderate effects and more subtle small effects are adequately captured. This is crucial when examining complex biological relationships, as even small physiological changes can have significant clinical implications or indicate early stages of adaptation or pathology [57,58].

### 2.8. Statistical Analysis

All statistical analyses were completed in R (version 4.4.1) [59]. Sex-based differences in baseline demographics and cardiorespiratory variables were assessed using chi-square tests for categorical data and unpaired *t*-tests with Cohen’s d effect sizes for continuous data. Cohen’s d thresholds included negligible (<0.2), small (0.2–0.5), moderate (0.5–0.8), and large (>0.8) [60,61].

For participants who did not complete the exercise test, missing VO_2max_ data were imputed using multiple imputation by chained equations with 10 permutations and 25 iterations per imputation, incorporating available data from EEG, fNIRS, and TCD, as well as sex, age, and concussion history [56]. Linear models were used to analyze baseline, peak, area under the curve (AUC), and relative and absolute increases. The independent variables included sex (nominal; ref: Female), concussion history (nominal; ref: Conc Hx-), relative VO_2_ (continuous), age (continuous), and the self-reported presence of mental health diagnoses and/or learning disabilities (nominal; ref: none). Data are displayed as means with 95% confidence intervals.

Temporal responses between imaging modalities were computed using Granger causality analysis [62,63]. Granger causality was employed to determine the directional interactions between neuronal signals (EEG time–frequency bands) and vascular responses (HbO, HbR, MCAv, and PCAv) [62,63]. This method determines whether one signal provides statistically significant predictive information about the future values of another signal, beyond what is explained by the latter’s own past values. This method is similar to, albeit less computationally intensive than, a transfer entropy approach, which quantifies directed information flow using information-theoretic measures and typically requires estimation of probability distributions from the data [64]. Vector autoregressive (VAR) models of order *p* were constructed for pairs of signals of interest (EEG-HbO, EEG-HbR, EEG-MCAv, and EEG-PCAv).

To test whether EEG activity Granger-caused vascular responses, the restricted model was defined as

$$y_{t}=\sum_{i=1}^{p}a_{i}y_{t-i}+\varepsilon_{t}^{\left(r\right)}$$

and the unrestricted model as

$$y_{t}=\sum_{i=1}^{p}a_{i}y_{t-i}+\sum_{i=1}^{p}b_{i}x_{t-i}+\varepsilon_{t}^{\left(u\right)}$$

where $y_{t}$ denotes the vascular signal at time t and $x_{t}$ denotes the EEG band power, $a_{i}$ is the autoregressive coefficients, $\varepsilon_{t}^{\left(r\right)}$ is the residual error term, and $b_{i}$ quantifies the influence of EEG activity on the vascular response.

Granger causality from EEG to the vascular signal was tested under the following condition:

$$H_{0}:{\;b}_{1}=b_{2}=\cdots =b_{p}=0$$

Reciprocal models were constructed to test whether vascular signals Granger-caused EEG activity:

$$x_{t}=\sum_{i=1}^{p}c_{i}x_{t-i}+\varepsilon_{t}^{\left(r\right)}$$

$$x_{t}=\sum_{i=1}^{p}c_{i}x_{t-i}+\sum_{i=1}^{p}d_{i}y_{t-i}+\varepsilon_{t}^{\left(u\right)}$$

with the null hypothesis

$$H_{0}:d_{1}=d_{2}=\cdots =d_{p}=0$$

Causality strength in both directions was assessed using an F-statistic comparing residual sum of squares between restricted and unrestricted models:

$$F=\frac{(RSS_{r}-RSS_{u})/p}{RSS_{u}/(T-2p-1)}$$

where $RSS_{r}$ and $RSS_{u}$ denote the residual sum of squares of the restricted and unrestricted models, respectively, while T represents the number of observations. A significantly lower prediction error in the unrestricted model indicated a directional predictive influence between modalities.

The Granger causality analysis was conducted for each individual’s block-averaged EEG, fNIRS, and TCD responses during the motor and visual tasks; however, these were divided into time bins to determine the predictive value of these signals during different phases of the tasks. For the Waldo task, time bins of −10–0, 0–10, 10–20, and 20–30 s were used based on stimulus onset with eyes open, while for finger tapping, time bins of −10–5, −5–0, 0–5, and 5–10 s were used based on the tapping onset. This was then converted into a percentage of participants who displayed a significant directional relationship within each time grouping. These were binned into quintiles of very weak (0–20%), weak (20–40%), moderate (40–60%), strong (60–80%), and very strong (80–100%) relationships. Alpha was set a priori at 0.05.

## 3. Results

### 3.1. Waldo Granger Causality Analysis

**Figure 2:**
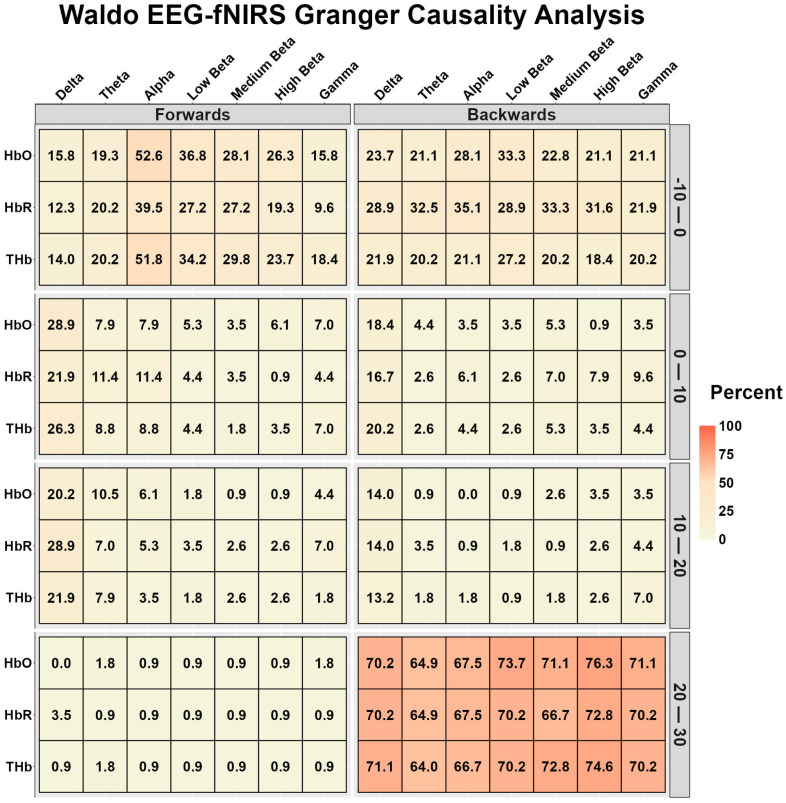
Bidirectional Granger causality analysis results between electroencephalography (EEG) and functional near-infrared spectroscopy (fNIRS) during the visual “Where’s Waldo?” task. The results are the percentage of participants whose data displayed a significant predictive value between waveforms, stratified into 10-s time bins (*n* = 113, 86 females and 27 males). EEG time–frequency bands were delta (0.5–4 Hz), theta (4–8 Hz), alpha (8–12 Hz), low beta (12–16 Hz), medium beta (16–20 Hz), high beta (20–30 Hz), and gamma (30–40 Hz). Oxygenated hemoglobin (HbO), deoxygenated hemoglobin (HbR), and total hemoglobin (THb).

Figure 2 displays the EEG-fNIRS and fNIRS-EEG temporal relationships during Waldo. During the 10 s preceding task onset, alpha displayed a moderate relationship with HbO and ThB, while the remaining variables displayed very weak to weak relationships (Figure 2). During time bins 0–10 and 10–20 s, very weak to weak relationships were identified (Figure 2). During the 20–30-s interval, EEG-fNIRS had very weak relationships, while fNIRS-EEG had strong relationships (Figure 2).

**Figure 3:**
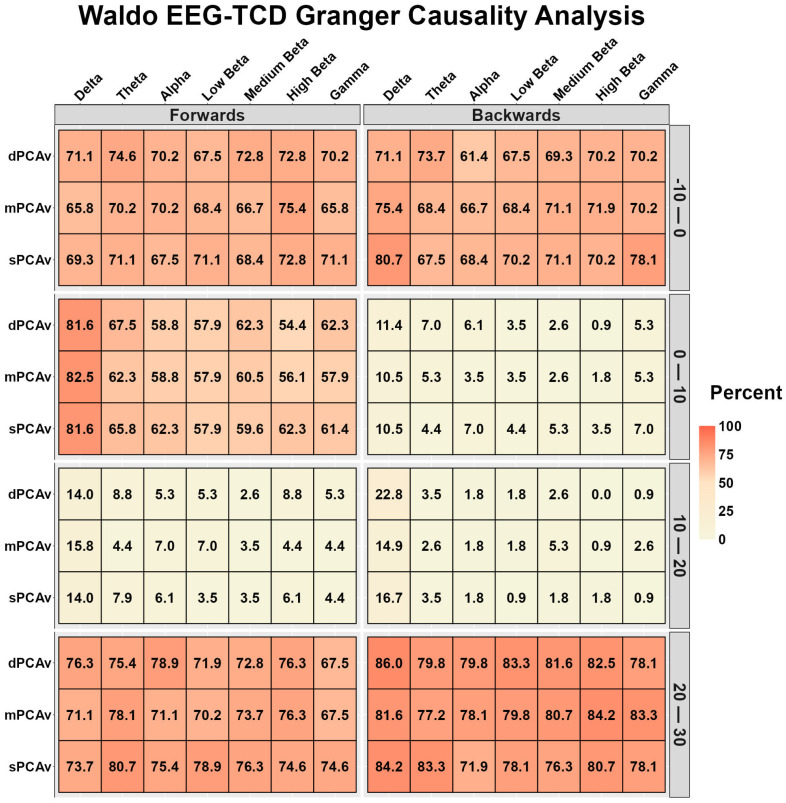
Bidirectional Granger causality analysis results between electroencephalography (EEG) and transcranial Doppler ultrasound (TCD) during the visual “Where’s Waldo?” task. The results are the percentage of participants whose data displayed a significant predictive value between waveforms, stratified into 10-s time bins (*n* = 113, 86 females and 27 males). EEG time–frequency bands are delta (0.5–4 Hz), theta (4–8 Hz), alpha (8–12 Hz), low beta (12–16 Hz), medium beta (16–20 Hz), high beta (20–30 Hz), and gamma (30–40 Hz). Diastolic posterior cerebral artery velocity (dPCAv), mean posterior cerebral artery velocity (mPCAv), systolic posterior cerebral artery velocity (sPCAv).

Figure 3 displays the EEG-TCD and TCD-EEG temporal relationships during Waldo. During the 10 s preceding task onset and 20–30 s after task onset, both displayed strong to very strong bidirectional relationships (Figure 3). During the 0–10 s after task onset, EEG-TCD displayed strong to very strong relationships, while the TCD-EEG relationships were very weak (Figure 3). The bidirectional relationships were very weak to weak during the 10–20 s after task onset (Figure 3).

**Figure 4:**
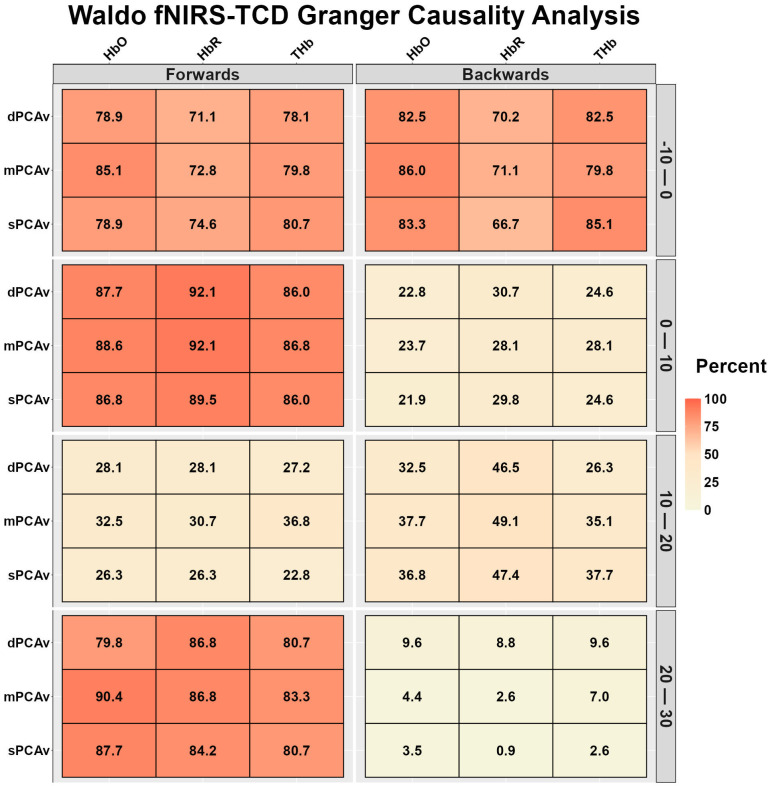
Bidirectional Granger causality analysis results between functional near-infrared spectroscopy (fNIRS) and transcranial Doppler ultrasound (TCD) during the visual “Where’s Waldo?” task. The results are the percentage of participants whose data displayed a significant predictive value between waveforms, stratified into 10-s time bins (*n* = 113, 86 females and 27 males). Oxygenated hemoglobin (HbO), deoxygenated hemoglobin (HbR), and total hemoglobin (THb), diastolic posterior cerebral artery velocity (dPCAv), mean posterior cerebral artery velocity (mPCAv), systolic posterior cerebral artery velocity (sPCAv).

Figure 4 displays the fNIRS-TCD and TCD-fNIRS temporal relationships during Waldo. The bidirectional relationships were strong to very strong during the 10 s preceding task onset (Figure 4). During 0–10 and 20–30 s after task onset, fNIRS-TCD displayed strong to very strong relationships, while the reverse TCD-fNIRS relationships during these times were very weak to weak (Figure 4). During the 10–20 s after task onset, TCD-fNIRS relationships displayed moderate relationships for HbR, while HbO, THb TCD-fNIRS and all fNIRS-TCD relationships were weak (Figure 4).

### 3.2. Finger Tapping Granger Causality Analysis

**Figure 5:**
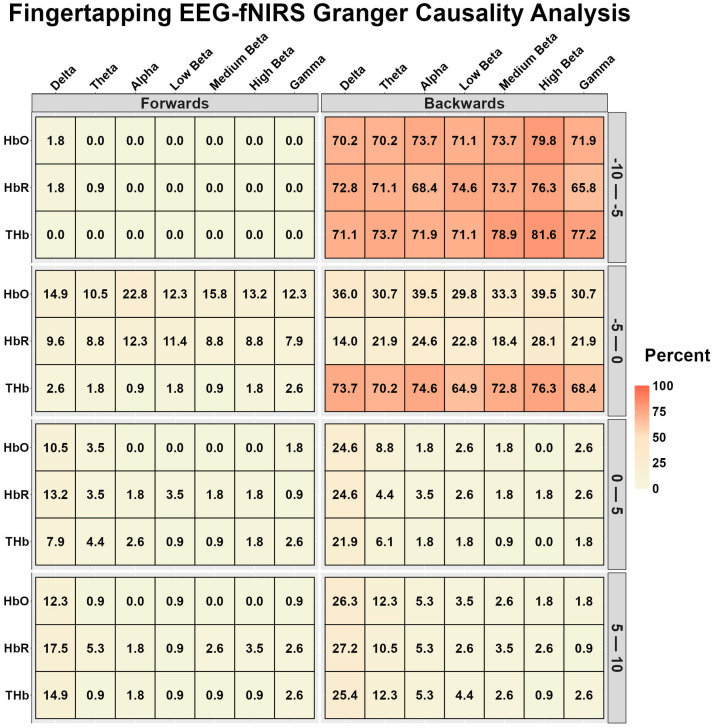
Bidirectional Granger causality analysis results between electroencephalography (EEG) and functional near-infrared spectroscopy (fNIRS) during the motor finger-tapping task. The results are the percentage of participants whose data displayed a significant predictive value between waveforms, stratified into 5-s time bins (*n* = 113, 86 females and 27 males). EEG time–frequency bands are delta (0.5–4 Hz), theta (4–8 Hz), alpha (8–12 Hz), low beta (12–16 Hz), medium beta (16–20 Hz), high beta (20–30 Hz), and gamma (30–40 Hz). Oxygenated hemoglobin (HbO), deoxygenated hemoglobin (HbR), and total hemoglobin (THb).

Figure 5 displays the EEG-fNIRS and fNIRS-EEG temporal relationships during finger tapping. Strong to very strong relationships were observed for fNIRS-EEG for all chromophores during the 15–20 s after task onset (5–10 s after tapping offset) (Figure 5) and THb in the preceding 5 s before task onset (Figure 5). However, all other relationships were weak to very weak (Figure 5).

**Figure 6:**
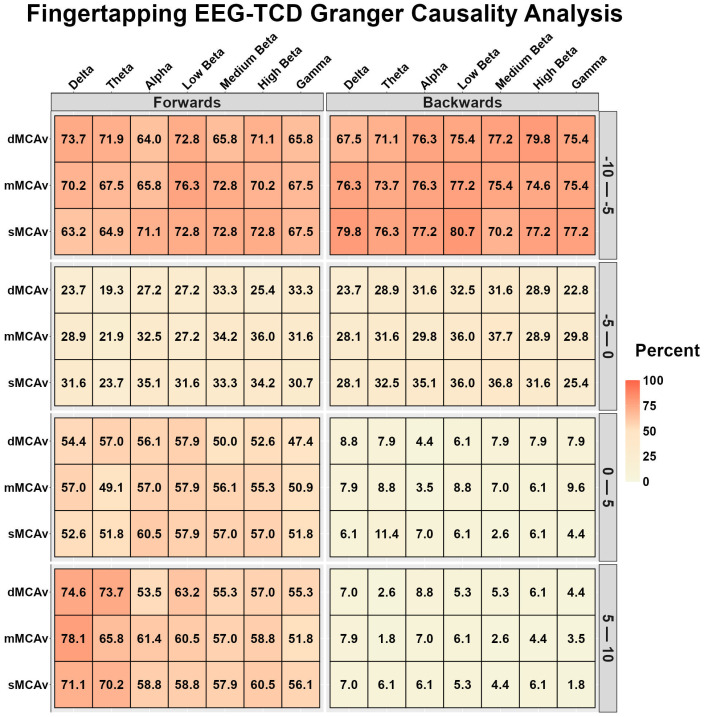
Bidirectional Granger causality analysis results between electroencephalography (EEG) and transcranial Doppler ultrasound (TCD) during the motor finger-tapping task. The results are the percentage of participants whose data displayed a significant predictive value between waveforms, stratified into 5-s time bins (*n* = 113, 86 females and 27 males). EEG time–frequency bands are delta (0.5–4 Hz), theta (4–8 Hz), alpha (8–12 Hz), low beta (12–16 Hz), medium beta (16–20 Hz), high beta (20–30 Hz), and gamma (30–40 Hz). Diastolic middle cerebral artery velocity (dMCAv), mean middle cerebral artery velocity (mMCAv), systolic middle cerebral artery velocity (sMCAv).

Figure 6 displays the EEG-TCD and TCD-EEG temporal relationships during finger tapping. During the 10–5 s preceding task onset, both bidirectional relationships were strong to very strong, while during the 5 s preceding task onset, both bidirectional relationships were very weak to weak (Figure 6). During finger tapping (0–10 s), the EEG-TCD relationships were weak to strong, while the TCD-EEG relationships were very weak (Figure 6).

**Figure 7:**
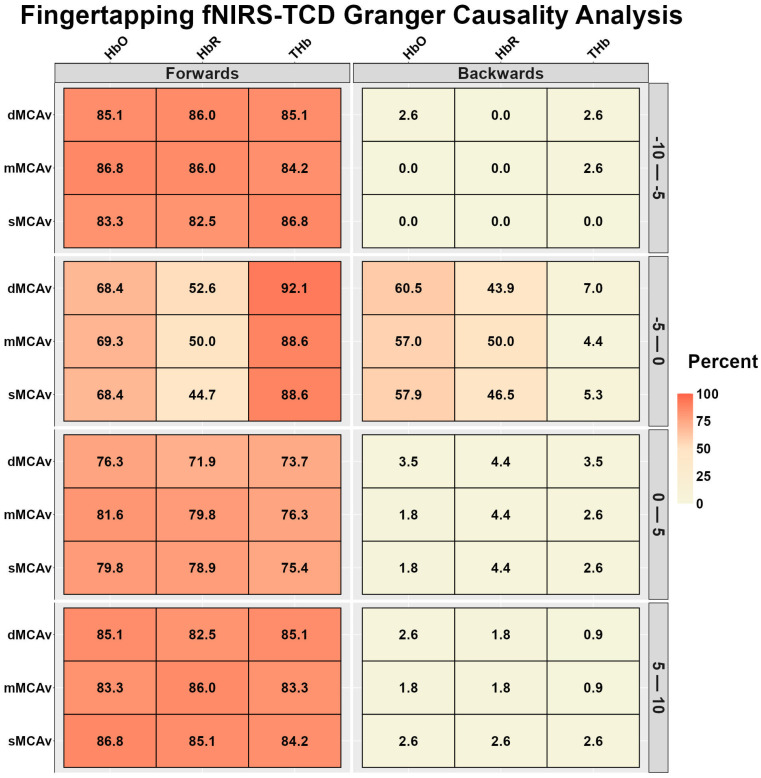
Bidirectional Granger causality analysis results between functional near-infrared spectroscopy (fNIRS) and transcranial Doppler ultrasound (TCD) during the motor finger-tapping task. The results are the percentage of participants whose data displayed a significant predictive value between waveforms, stratified into 5-s time bins (*n* = 113, 86 females and 27 males). Oxygenated hemoglobin (HbO), deoxygenated hemoglobin (HbR), and total hemoglobin (THb).

Figure 7 displays the fNIRS-TCD and TCD-fNIRS temporal relationships during finger tapping. During the 10–5 s preceding task onset, the fNIRS-TCD relationships were very strong, while the TCD-fNIRS relationships were very weak (Figure 7). During the 5 s preceding task onset, fNIRS-TCD THb displayed very strong, HbO displayed strong, and HbR displayed moderate relationships with MCAv (Figure 7); however, the TCD-fNIRS relationships were very weak for THb, moderate to strong for HbO, and moderate for HbR (Figure 7). During the task, the fNIRS-TCD relationships were strong to very strong, while the TCD-fNIRS relationships were very weak (Figure 7).

### 3.3. Transcranial Doppler Ultrasound Hemodynamic Response

For the Waldo task, no differences were noted in the PCAv hemodynamic responses analysis for sex (all *p* > 0.078), concussion history (all *p* > 0.169), age (all *p* > 0.054), relative VO_2max_ (*p* > 0.320), or the mental health/learning disability composite (all *p* > 0.067) (Table 2). For finger tapping, compared to females, males had lower baseline and peak MCAv metrics across the cardiac cycle (all *p* < 0.001) (Table 2). No differences in NVC metrics were noted between those with and without a history of concussion (all *p* > 0.095) (Table 2). Age was negatively associated with baseline and peak MCAv across the cardiac cycle (all *p* < 0.001), in addition to a larger mean and systolic relative increase and systolic absolute increase in MCAv (all *p* < 0.004) (Table 2). A greater relative VO_2max_ was associated with higher baseline and peak MCAv across the cardiac cycle (all *p* < 0.049), excluding peak mean MCAv (*p* = 0.079) (Table 2). No associations were found between the mental health and learning disability composite and any MCAv metric (Table 2).

### 3.4. Functional Near-Infrared Spectroscopy Hemodynamic Response

For the Waldo task, no differences were noted across the three chromophore hemodynamic response analyses for sex (all *p* > 0.084), concussion history (all *p* > 0.240), age (all *p* > 0.364), relative VO_2max_ (*p* > 0.115), or the mental health/learning disability composite (all *p* > 0.321) (Table 3). For finger tapping, males had a greater baseline HbR (*p* = 0.048) and ThB (*p* = 0.021) concentrations; however, no other sex differences were noted (*p* > 0.070) (Table 3). No other associations were found for concussion history (all *p* > 0.220), age (all *p* > 0.196), relative VO_2max_ (*p* > 0.121), or the mental health/learning disability composite (all *p* > 0.283) (Table 3).

### 3.5. Electroencephalography Neurodynamic Response

For the Waldo task, no differences were noted across both alpha and low beta power time–frequency responses for sex (all *p* > 0.237), concussion history (all *p* > 0.287), age (all *p* > 0.191), relative VO_2max_ (*p* > 0.146), or the mental health/learning disability composite (all *p* > 0.494) (Table 4). For finger tapping, age was associated with a greater low beta AUC (*p* = 0.017); however, no associations were found for sex (*p* > 0.237), concussion history (all *p* > 0.273), age (all *p* > 0.072), relative VO_2max_ (*p* > 0.190), or the mental health/learning disability composite (all *p* > 0.384) (Table 4).

## 4. Discussion

The purpose of the present study was to assess the NVC response concurrently at the neural, microvascular, and intracranial cerebral artery levels in a large cohort of young adults. While the NVC response describes the coupling of blood flow to neurovascular metabolism, the three assessment techniques employed in this investigation have not historically been measured simultaneously. The study revealed many novel finCdings. Firstly, the Granger causality analysis demonstrated that neuronal and microvascular activities were unidirectionally predictive of arterial changes during the initial 10 s of both visual- and motor-based tasks. Moreover, when a steady state was achieved, a bidirectional predictive relationship was identified, suggesting that different regions of the brain communicated to maintain hemostasis around the new set-point. Furthermore, the EEG-fNIRS Granger causality analyses demonstrated minimal relationships between variables, potentially due to the insufficient sampling of the fNIRS (3.90 Hz; 256 ms) to detect the immediate neuronal–microvascular signaling. Finally, the NVC response metrics, characterized through the absolute increase, relative increase, and AUC, were minimally influenced by sex, concussion history, cardiorespiratory fitness levels, age during the young adulthood period, and mental health disorders/learning disabilities. Collectively, these results demonstrate a regional signaling propagation from the capillaries up the arterial tree to influence blood delivery; however, the introduction of higher temporal resolution fNIRS devices would enable a more intricate neuronal–microvascular–arterial quantification.

To date, the relative contributions of the known mechanisms that moderate the NVC response are debated [1,2,4]. The NVC response is typically described as an intracranial arterial response that lags behind an increase in neural metabolism, because of both an increase in energy substrate consumption and increased accumulation of metabolic waste [1,2,4]. The periods of transition from neurological rest to activation are of particular interest for investigating the mechanistic underpinnings of the known NVC response. The present analysis demonstrated that the active neurological response to the visual “Where’s Waldo?” and motor finger-tapping paradigms, quantified using EEG, was predictive of the vascular response assessed by TCD (Figure 2 and Figure 5). This finding provides objective evidence to support the proposed theory of neural activation initiating large-scale compensatory changes in regional CBF. While this association was expected, the unique capacity of the present study is the evaluation of the influence of the microvasculature, which represents the missing causal link in nutrient delivery from the microvasculature to the neuron, as well as between the microvasculature and the upstream arteries. The fNIRS-TCD relationship demonstrated similar predictive capabilities, albeit stronger during the visual task (Figure 3 and Figure 6).

There is evidence for a number of compensatory and overlapping mechanisms that influence the dilation of capillaries and arterioles. For example, a meta-analysis by Hosford and Gourine [65] on the mediators of the NVC response identified that neural nitric oxide (NO) synthase reduced the NVC response by 64%, while other neurochemicals explained some of the response. The authors concluded that one-third of the signaling was unaccounted for [65]. Furthermore, NO has been proposed to potentially cause dilation of the capillaries via pericyte activation and adenosine accumulation, arterioles via cyclic guanosine monophosphate (cGMP) and soluble guanylate cyclase pathways, and arteries via vascular smooth muscle cells [66,67,68]. In neurons, NO is produced via neural NO synthase, activated by neural depolarization, which then acts on pericytes to mediate capillary dilation [68]. NO is also produced by endothelial cells which then act on the vascular smooth muscle cells ‘underneath’ them via cGMP. Hence, NO is a potent agent for the NVC response at all levels of the vasculature, though via different mechanisms. Finally, the release of potassium during neuronal activity hyperpolarizes endothelial cells and triggers retrograde vasodilation through endothelial inward rectifier potassium channels [69,70]. This upstream propagation via ionic currents through gap junctions between endothelial cells and myoendothelial junctions with smooth muscle cells ensures coordinated dilation of upstream vessels [71,72]. Hence, the NVC response is driven by a complex interplay between both neuronal and vascular signaling molecules.

The result of the Granger analyses indicated that, as previously suggested, the microvasculature responds to local changes in neural metabolism, driving upstream regulation of the intracranial arteries to maintain homeostatic delivery of nutrients to the neurons [1,2,4]. There are three potential explanations/hypotheses for the coordinated response in the microvasculature and intracranial arteries. The first suggests that the neural control of endothelial cells in the intracranial arteries is activated during periods of microvascular dilation, causing a compensatory increase in flow to areas of increased microvascular flow [73]. Conversely, microvascular dilation in response to neural activity may result in a regional decrease in microvascular pressure, increasing the perfusion pressure in the upstream intracranial arteries, which would increase CBv [1,2,4]. Lastly, direct innervation of the vascular endothelium may co-occur with direct innervation of the microvasculature, but with different timing, rendering the microvascular response predictive of the TCD response, despite the absence of a direct causal mechanism. For example, larger cerebral arteries receive sympathetic innervation from the superior cervical ganglion, while cortical microvessels are innervated by noradrenergic, serotonergic, cholinergic, and GABAergic neurons from subcortical and local cortical sources. This suggests that both vascular compartments are directly influenced by neuronal activity through distinct pathways and temporal patterns [25,74].

To the authors’ surprise, the Granger causality analysis observed very little predictive association between the EEG-derived frequency band response and the microvascular responses to both tasks (Figure 1 and Figure 4). Theoretically, one would expect this to be similar to the EEG-TCD comparison but temporally quicker. Hence, the lack of findings could be in part explained by the fNIRS device sampling every 256 ms, missing the near-instantaneous signaling of the EEG-fNIRS relationship. Evidence for this proposition is the similar pattern of predictiveness for the EEG-TCD and fNIRS-TCD (Figure 2, Figure 3, Figure 5 and Figure 6), with arterial changes occurring ~1–2 s post-stimulus onset [5]. A sampling rate of 3.90 Hz would be sufficient to capture the microvasculature-to-arterial delay (Figure 3 and Figure 6); however, the rapid coupling of the microvasculature-to-neurological activity may have been missed due to the lower temporal resolution of the microvascular assessment (fNIRS). Finally, the frequency band assessments may be too non-specific to capture the localized response to the NVC task by the neural areas driving the NVC response [5]. Interestingly, delta band activity demonstrated stronger Granger relationships with the vascular signals compared with other EEG frequency bands. This may reflect the closer alignment between slower cortical oscillations and hemodynamic fluctuations, as low-frequency neural activity has previously been linked to vascular and metabolic processes underlying NVC [75].

After ~15–25 s with the NVC response, a new homeostatic set point is achieved (75). The feedback loop then becomes more prominent, given that the regulation requires smaller adjustments to maintain this new steady state [4]. The Granger causality relationship between variables demonstrated a bidirectional predictive relationship showing the crosstalk between signals to maintain homeostasis (Figure 1, Figure 2, Figure 3, Figure 4, Figure 5 and Figure 6). This relationship was more apparent in the Waldo task compared to the finger-tapping task, potentially due to the visual task eliciting greater neuronal/hemodynamic differences between the rest and active sections of each task (Figure 1, Figure 2, Figure 3, Figure 4, Figure 5 and Figure 6). This could also be attributable to regional differences in the NVC response, which warrants further consideration. Finally, the fact that bidirectional relationships were present between EEG-fNIRS signals during the steady-state portions of the tasks provides further support for the notion that the sampling rate was insufficient during the initial task activation.

There has been a recent emphasis on improving the generalizability and understanding of sex differences in physiological research by including male and female participants [76,77,78]. There are known sex differences in intracranial arterial CBv [22,79], cerebral hemoglobin concentration [80], and neurological function at rest [10,11], as well as in evoked potentials [81]. The present study detected a sex difference in baseline MCAv measures and in the peak MCAv response to the finger-tapping stimulus. The difference in the MCA NVC response is likely a byproduct of the elevated resting CBv detected in the female participants compared to the males, rather than a purely mechanistic difference in how the NVC response occurs between biological sexes (Table 2). Baseline sex differences were also noted for fNIRS, with males having higher THb and HbR concentrations compared to females during the finger-tapping task (Table 3). While this may point to hemodynamic sex differences within the microvessels, it could also be the result of a type I error. Nonetheless, it is established that males have a greater hematocrit concentration, leading to greater absolute concentrations of HbO, HbR, and THb [82]. As this investigation utilized a continuous-wave device, only relative concentrations were discernible. Hence, the quantification of relative concentrations would dampen the known absolute concentration differences. Finally, the current investigation highlighted a lack of sex-based differences in the alpha and low beta power-band frequencies. This could potentially be attributable to the task states washing out potential resting-state differences. Moreover, the only investigations to date that have utilized the “Where’s Waldo?” paradigm with EEG have been conducted by the current research group.

Concussion is characterized in part by a traumatic force impacting brain function yet often shows a negative result on a standard neuroimaging evaluation, such as computed tomography or magnetic resonance scan [83,84]. Nonetheless, acute functional alterations have been noted in the immediate days following concussion, which has prompted mainstream concern regarding the long-term neurological consequences of concussion [9,85,86]. This has further been amplified given the perceived potential associations with Alzheimer’s disease and related dementias [87], mild cognitive decline with age [88], depression and suicidality [89], and chronic traumatic encephalopathy [90]; however, the direct associations have commonly been overstated [91,92,93,94,95,96,97]. Previous research focused on the consequences of concussion includes the use of EEG [81,98,99,100,101], fNIRS [102,103,104,105] TCD [9,14,106,107,108], and fMRI [109,110,111,112,113,114] to assess impaired functional differences following concussion, with mixed findings. The current investigation noted no differences in those with and without a history of concussion; however, it is important to note that the present investigation focused on the NVC response. Therefore, future work into resting-state, cerebral autoregulation, and cerebrovascular reactivity domains is warranted to fully elucidate the long-term ramifications of concussion on cerebrovascular regulation.

While cerebral perfusion declines with age, which is associated with declining cognition, high physical fitness slows the decline in cerebral perfusion by roughly 10 years [17] and attenuates the odds of age-related cognitive decline by 41% [115]. The findings in Table 2 highlight that age and cardiorespiratory fitness had the largest influence on the intracranial arteries, with baseline and peak MCAv declining at a greater rate with age across young adulthood. However, fitness was associated with a higher MCAv, illustrating and confirming prior research showing that exercise in young adulthood is beneficial for augmenting cerebral perfusion. Despite these findings, the NVC response quantified through the absolute/relative increase and AUC was only nominally influenced. While fitness improves overall CBv indexes, the NVC response appears to be intact across the young adult spectrum. Previous work by Koep et al. [22] highlighted an age-by-sex interaction with a positive increasing relationship between PCAv and age in females only. This previous investigation was completed across all adulthood stages (18–85 years), while the current investigation was restricted to young adulthood (18–40 years). Hence, it appears that cardiorespiratory fitness and age may not need to be included as confounding factors within statistical models of NVC in young adulthood; however, if researchers are examining absolute CBv metrics, fitness is an important variable to include in statistical models when examining MCAv/PCAv metrics.

Finally, the linear model analyses found minimal influence of the mental health disorders/learning disability composite on the NVC response across all three imaging domains. A limitation of the included composite was that it grouped all participants together with a physician-diagnosed disorder/disability across several domains (e.g., major depressive disorder, anxiety, attention deficit hyperactivity disorder) as the study was underpowered to investigate each domain independently. Nonetheless, the collective use of these variables has been under-examined to date, and further work using scoring on continuous-based questionnaires (e.g., Patient Health Questionnaire-9, Generalized Anxiety Disorder-7) may be more sensitive than nominal groupings.

The primary limitation of the present investigation is the inherent difference in temporal resolution across the multimodal neuroimaging techniques employed. EEG data were acquired at millisecond-scale temporal resolution, reflecting rapid neuronal dynamics, whereas TCD and fNIRS capture hemodynamic responses at 100 and 3.90 Hz, respectively. These device differences in temporal properties required temporal alignment procedures, including averaging, resampling, and interpolation, to enable cross-modal comparison and Granger causality analyses. While this limits direct comparability of absolute timing between neuronal and vascular responses and may potentially lead to biased timing estimates, there is currently no commercially available platform that integrates EEG, fNIRS, and TCD within a unified acquisition system for clinical or research use. Therefore, the present investigation relied on independent acquisition systems that required post hoc synchronization and temporal alignment across modalities. Although this introduces inherent methodological challenges related to sampling frequency and timing precision, this is the first large-scale investigation to simultaneously integrate EEG, fNIRS, and TCD to examine NVC dynamics in humans, and the methodological trade-offs associated with multimodal synchronization represent a necessary step toward comprehensive characterization of neuronal–vascular interactions in humans.

Another limitation was the number of participants who did not complete the VO_2max_ testing. However, to reduce the influence of bias, missing data were predicted using multiple imputation by chained equations models that used all of the NVC and demographic data. This approach has been demonstrated to be highly robust for accounting for missing data [56,116,117]. There is some evidence that hormonal contraceptives may influence cerebrovascular function, which was not considered in the present study. However, to minimize this influence, females were tested during days 3–10 of their menstrual cycle, when hormones are known to be at their lowest levels [118,119].

To combine the assessment modalities, the spatial resolution of fNIRS and EEG was limited by the locations on the multimodal cap (i.e., centralized to the midline of the head). Thus, regional differences in the NVC response were not compared in the present study; however, pilot work demonstrated the largest responses within the occipital and motor regions for the visual- and motor-based tasks, respectively. Furthermore, while the study aimed to quantify the coupling of neurological activity with cerebral blood flow, intracranial arterial blood flow could not be directly assessed. CBv was a surrogate measure for flow, given that the diameter of the intracranial vessels could not be assessed. Thus, the influences of the endothelial NVC response were not assessed.

## 5. Conclusions

This study sought to isolate the feedforward and feedback loops of the NVC response in humans using a concurrent multimodal imaging approach of EEG-fNIRS-TCD. The Granger causality findings demonstrated a clear unidirectional feedforward mechanism between the EEG-TCD and fNIRS-TCD responses; however, no associations were found during task onset for the EEG-fNIRS relationship. This could be due to the lower temporal resolution of fNIRS (3.90 Hz), which was not sufficient to detect the neuronal-to-microvascular signaling that could occur within 256 ms of task onset. Future work utilizing fNIRS devices with greater temporal resolution and/or different analytical methods may be able to tease apart the precise underpinnings between local neuronal-vascular regions. Finally, aside from known arterial influences of biological sex, age, and cardiorespiratory fitness on CBv, these factors, in addition to concussion history and mental health/learning disabilities, had minimal influence on the NVC response across all three imaging domains. This increases the generalizability of previous NVC research findings, suggesting that future investigations may not need to consider these as confounding influences to control within the study design and/or statistical analyses.

## Figures and Tables

**Table 1:** Demographics of participants (*n* = 113).

Variable	Total (*n* = 113)	Females (*n* = 86)	Males (*n* = 27)	Comparison
Age (Years)	24.8 (4.5)	24.9 (4.6)	24.7 (4.0)	*p* = 0.833, *d* = 0.05 [negligible]
Height (cm)	168.7 (9.3)	165.8 (7.7)	178.2 (7.3)	*p* < 0.001, *d* = 1.66 [large]
Weight (kg)	68.7 (11.8)	65.5 (9.6)	79.0 (12.7)	*p* < 0.001, *d* = 1.21 [large]
BMI (kg/m^2^)	24.0 (2.9)	23.8 (2.8)	24.8 (3.3)	*p* = 0.149, *d* = 0.34 [small]
Calgary Pressure (mmHg)	660.0 (61.7)	665.9 (5.6)	641.5 (125.9)	*p* = 0.324, *d* = 0.27 [small]
Temperature (°C)	22.3 (2.8)	22.0 (0.3)	22.0 (0.2)	*p* = 0.633, *d* = 0.10 [negligible]
Humidity (%)	22.1 (11.9)	23.0 (12.2)	19.3 (10.5)	*p* = 0.137, *d* = 0.32 [small]
Absolute VO_2max_	3.4 (0.9)	2.9 (0.6)	4.3 (0.8)	*p* < 0.001, *d* = 2.06 [large]
Missing	56	48 (55.8%)	8 (29.6%)	
Relative VO_2max_	43.2 (8.1)	40.1 (7.5)	49.1 (7.6)	*p* < 0.001, *d* = 1.22 [large]
Concussion History				χ^2^ = 1.04, *p* = 0.308
Yes	35 (31.0%)	24 (27.9%)	11 (40.7%)	
No	78 (69.0%)	62 (72.1%)	16 (59.3%)	
Number of Previous Concussion				χ^2^ = 8.46, *p* = 0.037
0	78 (69.0%)	62 (72.1%)	16 (59.3%)	
1	24 (21.2%)	17 (19.8%)	7 (25.9%)	
2	5 (4.4%)	5 (5.8%)	0 (0.0%)	
3+	6 (5.3%)	2 (2.4%)	4 (14.8%)	
Mental Health				χ^2^ = 0.96, *p* = 0.328
Yes	22 (19.5%)	19 (22.1%)	3 (11.1%)	
No	91 (80.5%)	67 (77.9%)	24 (88.9%)	
Learning Disability				χ^2^ = 0.38, *p* = 0.540
Yes	19 (16.8%)	16 (18.6%)	3 (11.1%)	
No	94 (83.2%)	70 (81.4%)	24 (88.9%)	
Degree				χ^2^ = 9.30, *p* = 0.098
High School Diploma	17 (15.0%)	11 (12.8%)	6 (22.2%)	
Some College, No Degree	15 (13.3%)	12 (14.0%)	3 (11.1%)	
Trade School	2 (1.8%)	2 (2.3%)	0 (0.0%)	
Bachelor’s Degree	47 (41.6%)	38 (44.2%)	9 (33.3%)	
Graduate Classes, No Degree	11 (9.7%)	5 (5.8%)	6 (22.2%)	
Graduate Degree	21 (18.6%)	18 (20.9%)	3 (11.1%)	
Ethnicity				χ^2^ = 3.64, *p* = 0.457
Asian	11 (9.7%)	8 (9.3%)	3 (11.1%)	
Hispanic	5 (4.4%)	4 (4.7%)	1 (3.7%)	
Indigenous	1 (0.9%)	1 (1.2%)	0 (0.0%)	
White	94 (83.2%)	72 (83.7%)	22 (81.5%)	
Mixed	2 (1.8%)	1 (1.2%)	1 (3.7%)	
Contraceptive Usage				
Natural	-	42 (45.7%)	**-**	
Hormonal IUD	-	33 (40.7%)	**-**	
Oral Contraceptive	-	11 (13.6%)	**-**	
“Where’s Waldo?” Physiological Values				
Systolic Blood Pressure	118.8 (16.4)	117.5 (14.6)	123.0 (21.4)	*p* = 0.238, *d* = 0.30 [small]
Mean Blood Pressure	80.1 (12.7)	79.2 (12.0)	83.1 (14.6)	*p* = 0.228, *d* = 0.29 [small]
Diastolic Blood Pressure	63.5 (11.4)	62.5 (10.8)	66.8 (12.8)	*p* = 0.133, *d* = 0.37 [small]
Heart Rate	72.6 (12.4)	73.9 (12.3)	68.2 (11.9)	*p* = 0.039, *d* = 0.47 [small]
End-Tidal Carbon Dioxide	36.7 (2.4)	36.4 (2.5)	37.7 (2.0)	*p* = 0.008, *d* = 0.58 [moderate]
Finger Tapping Physiological Values				
Systolic Blood Pressure	122.2 (15.8)	120.5 (15.1)	128.6 (17.1)	*p* = 0.046, *d* = 0.51 [moderate]
Mean Blood Pressure	81.7 (12.0)	80.0 (11.2)	87.6 (13.4)	*p* = 0.019, *d* = 0.61 [moderate]
Diastolic Blood Pressure	63.3 (10.6)	61.3 (9.7)	71.1 (10.4)	*p* = 0.001, *d* = 0.98 [large]
Heart Rate	71.8 (12.0)	72.7 (12.2)	69.1 (11.4)	*p* = 0.168, *d* = 0.30 [small]
End-Tidal Carbon Dioxide	36.8 (2.5)	36.5 (2.4)	37.7 (2.3)	*p* = 0.030, *d* = 0.49 [small]

Comparisons were completed using chi-square tests on categorical data and unpaired *t*-tests with Cohen’s *d* effect sizes on continuous data. Centimeters (cm), kilograms (kg), maximal oxygen uptake (VO_2max_), intrauterine device (IUD).

**Table 2:** Transcranial Doppler hemodynamic response function linear mixed effect models coefficients and 95% confidence intervals in 113 participants (77% female) during visual- and motor-based neurovascular coupling tasks.

Task	Metric	Variable	Baseline	Peak	Absolute Increase	Relative Increase	AUC
Waldo	Diastole PCAv	Sex (F–Ref)	−1.3 (−5.4, 2.9); *p* = 0.540	−1.8 (−6.4, 2.9); *p* = 0.452	−0.7 (−2.6, 1.2); *p* = 0.492	−158.5 (−989.5, 672.5); *p* = 0.706	−8.0 (−60.9, 44.9); *p* = 0.765
		Conc Hx (No–Ref)	1.0 (−2.4, 4.4); *p* = 0.562	−0.1 (−3.8, 3.7); *p* = 0.966	−0.9 (−2.6, 0.7); *p* = 0.262	−255.6 (−942.6, 431.4); *p* = 0.462	−29.5 (−71.8, 12.7); *p* = 0.169
		Age	−0.2 (−0.5, 0.1); *p* = 0.250	−0.1 (−0.4, 0.2); *p* = 0.519	0.1 (−0.1, 0.2); *p* = 0.358	−16.0 (−78.3, 46.2); *p* = 0.610	2.1 (−1.6, 5.9); *p* = 0.262
		Relative VO_2max_	−0.1 (−0.3, 0.1); *p* = 0.320	−0.1 (−0.3, 0.1); *p* = 0.431	0.0 (−0.1, 0.1); *p* = 0.538	−8.9 (−53.9, 36.1); *p* = 0.695	0.2 (−2.7, 3.0); *p* = 0.915
		Mental Health	−1.9 (−5.6, 1.7); *p* = 0.297	−1.0 (−4.9, 3.0); *p* = 0.630	0.9 (−0.8, 2.6); *p* = 0.304	−48.1 (−777.8, 681.6); *p* = 0.896	19.8 (−24.5, 64.0); *p* = 0.377
	Mean PCAv	Sex (F–Ref)	−3.9 (−8.8, 1.0); *p* = 0.114	−4.3 (−9.9, 1.3); *p* = 0.131	−0.9 (−3.2, 1.5); *p* = 0.462	0.6 (−9.0, 10.1); *p* = 0.909	−9.3 (−71.1, 52.4); *p* = 0.765
		Conc Hx (No–Ref)	1.1 (−2.9, 5.1); *p* = 0.596	0.4 (−4.3, 5.2); *p* = 0.857	−0.7 (−2.6, 1.3); *p* = 0.506	−3.9 (−11.8, 3.9); *p* = 0.324	−25.3 (−74.2, 23.5); *p* = 0.306
		Age	−0.3 (−0.6, 0.1); *p* = 0.154	−0.2 (−0.6, 0.2); *p* = 0.408	0.1 (−0.1, 0.2); *p* = 0.541	0.2 (−0.5, 0.9); *p* = 0.568	1.8 (−2.5, 6.2); *p* = 0.407
		Relative VO_2max_	−0.0 (−0.3, 0.2); *p* = 0.739	−0.1 (−0.3, 0.2); *p* = 0.669	0.1 (−0.1, 0.2); *p* = 0.418	−0.1 (−0.6, 0.4); *p* = 0.767	−0.1 (−3.4, 3.2); *p* = 0.946
		Mental Health	−2.5 (−6.6, 1.7); *p* = 0.244	−2.0 (−7.1, 3.0); *p* = 0.428	0.7 (−1.4, 2.7); *p* = 0.515	3.0 (−5.3, 11.3); *p* = 0.473	14.6 (−36.9, 66.2); *p* = 0.574
	Systole PCAv	Sex (F–Ref)	−6.1 (−12.9, 0.7); *p* = 0.078	−5.2 (−13.1, 2.8); *p* = 0.198	−0.1 (−2.9, 2.7); *p* = 0.946	0.3 (−4.8, 5.3); *p* = 0.912	−4.9 (−72.6, 62.9); *p* = 0.887
		Conc Hx (No–Ref)	1.6 (−4.1, 7.3); *p* = 0.577	0.9 (−5.7, 7.5); *p* = 0.796	−0.8 (−3.1, 1.5); *p* = 0.504	−2.2 (−6.4, 2.0); *p* = 0.297	−23.9 (−80.1, 32.3); *p* = 0.401
		Age	−0.5 (−1.0, 0.0); *p* = 0.054	−0.4 (−0.9, 0.2); *p* = 0.248	0.1 (−0.1, 0.3); *p* = 0.296	0.3 (−0.1, 0.6); *p* = 0.155	2.8 (−2.2, 7.8); *p* = 0.271
		Relative VO_2max_	0.0 (−0.3, 0.4); *p* = 0.805	−0.2 (−0.6, 0.3); *p* = 0.465	−0.1 (−0.2, 0.1); *p* = 0.368	0.1 (−0.2, 0.3); *p* = 0.608	−1.2 (−4.8, 2.4); *p* = 0.501
		Mental Health	−4.9 (−10.9, 1.1); *p* = 0.109	−4.7 (−11.7, 2.3); *p* = 0.185	0.6 (−1.8, 3.0); *p* = 0.601	4.1 (−0.3, 8.5); *p* = 0.067	20.3 (−38.8, 79.4); *p* = 0.497
Finger Tapping	Diastole MCAv	Sex (F–Ref)	**−10.3 (−15.1, −5.4); *p* < 0.001**	**−10.3 (−14.9, −5.6); *p* < 0.001**	−0.2 (−1.1, 0.6); *p* = 0.570	0.2 (−1.5, 1.9); *p* = 0.820	−0.5 (−7.4, 6.5); *p* = 0.892
		Conc Hx (No–Ref)	2.6 (−1.4, 6.5); *p* = 0.201	2.8 (−1.2, 6.8); *p* = 0.167	0.2 (−0.5, 0.9); *p* = 0.540	0.2 (−1.2, 1.7); *p* = 0.758	1.2 (−4.7, 7.2); *p* = 0.680
		Age	**−0.8 (−1.2, −0.4); *p* < 0.001**	**−0.8 (−1.2, −0.5); *p* < 0.001**	−0.0 (−0.1, 0.1); *p* = 0.755	0.1 (−0.0, 0.2); *p* = 0.132	0.2 (−0.4, 0.7); *p* = 0.552
		Relative VO_2max_	**0.3 (0.0, 0.5); *p* = 0.048**	**0.3 (0.1, 0.5); *p* = 0.013**	0.0 (−0.0, 0.1); *p* = 0.753	−0.0 (−0.1, 0.1); *p* = 0.980	0.1 (−0.2, 0.5); *p* = 0.509
		Mental Health	2.0 (−2.2, 6.2); *p* = 0.341	2.6 (−1.6, 6.9); *p* = 0.222	0.3 (−0.4, 1.1); *p* = 0.354	0.6 (−0.9, 2.2); *p* = 0.434	1.6 (−4.6, 7.8); *p* = 0.609
	Mean MCAv	Sex (F–Ref)	**−14.7 (−21.2, −8.2); *p* < 0.001**	**−14.6 (−21.1, −8.0); *p* < 0.001**	−0.3 (−1.1, 0.5); *p* = 0.496	0.9 (−4.1, 6.0); *p* = 0.714	−1.0 (−8.5, 6.5); *p* = 0.794
		Conc Hx (No–Ref)	4.5 (−0.8, 9.9); *p* = 0.095	4.4 (−1.1, 9.9); *p* = 0.113	0.2 (−0.5, 0.9); *p* = 0.636	−0.2 (−4.5, 4.0); *p* = 0.915	1.7 (−4.7, 8.2); *p* = 0.592
		Age	**−1.1 (−1.5, −0.6); *p* < 0.001**	**−1.1 (−1.5, −0.6); *p* < 0.001**	0.0 (−0.0, 0.1); *p* = 0.529	**1.2 (0.8, 1.6); *p* < 0.001**	0.2 (−0.3, 0.8); *p* = 0.409
		Relative VO_2max_	**0.4 (0.0, 0.8); *p* = 0.049**	0.3 (−0.0, 0.7); *p* = 0.079	−0.0 (−0.0, 0.0); *p* = 0.942	−0.3 (−0.5, 0.0); *p* = 0.060	0.1 (−0.3, 0.5); *p* = 0.541
		Mental Health	2.8 (−2.9, 8.4); *p* = 0.333	3.1 (−2.7, 8.9); *p* = 0.286	0.3 (−0.5, 1.0); *p* = 0.477	−1.7 (−6.2, 2.8); *p* = 0.460	0.7 (−6.1, 7.5); *p* = 0.836
	Systole MCAv	Sex (F–Ref)	**−18.8 (−27.8, −9.9); *p* < 0.001**	**−18.8 (−27.6, −10.0); *p* < 0.001**	−0.2 (−1.1, 0.7); *p* = 0.609	0.2 (−1.3, 1.7); *p* = 0.814	−0.7 (−8.4, 7.1); *p* = 0.864
		Conc Hx (No–Ref)	6.2 (−1.4, 13.8); *p* = 0.107	6.0 (−1.6, 13.6); *p* = 0.122	−0.1 (−0.9, 0.6); *p* = 0.742	−0.4 (−1.7, 0.9); *p* = 0.564	0.9 (−5.7, 7.4); *p* = 0.795
		Age	**−1.5 (−2.2, −0.8); *p* < 0.001**	**−1.4 (−2.1, −0.7); *p* < 0.001**	**0.1 (0.0, 0.2); *p* = 0.004**	**0.4 (0.3, 0.5); *p* < 0.001**	0.4 (−0.2, 1.0); *p* = 0.150
		Relative VO_2max_	**0.5 (0.0, 1.0); *p* = 0.038**	**0.5 (0.0, 1.0); *p* = 0.039**	−0.0 (−0.1, 0.0); *p* = 0.223	−0.1 (−0.2, 0.0); *p* = 0.051	−0.0 (−0.5, 0.4); *p* = 0.833
		Mental Health	3.5 (−4.5, 11.6); *p* = 0.385	3.7 (−4.4, 11.7); *p* = 0.370	0.0 (−0.8, 0.8); *p* = 0.990	−0.5 (−1.9, 0.9); *p* = 0.491	−0.4 (−7.3, 6.5); *p* = 0.908

Significant values are displayed in bold font. Area under the curve (AUC), posterior cerebral artery velocity (PCAv), middle cerebral artery velocity (MCAv), concussion history (Conc Hx), positive (+), negative (−), maximal oxygen update (VO_2max_).

**Table 3:** Functional near-infrared spectroscopy hemodynamic response function linear mixed effect models coefficients and 95% confidence intervals in 113 participants (77% female) during visual- and motor-based neurovascular coupling tasks.

Task	Chromophore	Variable	Baseline	Peak	Absolute Increase	AUC
Waldo	Occipital HbO	Sex (F–Ref)	1.8 × 10^−7^ (−5.0 × 10^−7^, 8.6 × 10^−7^); *p* = 0.606	−4.5 × 10^−7^ (−2.2 × 10^−6^, 1.3 × 10^−6^); *p* = 0.610	−6.2 × 10^−7^ (−2.9 × 10^−6^, 1.7 × 10^−6^); *p* = 0.601	−6.0 × 10^−6^ (−3.1 × 10^−5^, 1.9 × 10^−5^); *p* = 0.628
		Conc Hx (No–Ref)	9.5 × 10^−8^ (−5.1 × 10^−7^, 7.0 × 10^−7^); *p* = 0.758	−5.4 × 10^−7^ (−2.0 × 10^−6^, 9.5 × 10^−7^); *p* = 0.475	−6.5 × 10^−7^ (−2.7 × 10^−6^, 1.4 × 10^−6^); *p* = 0.527	−3.0 × 10^−6^ (−2.4 × 10^−5^, 1.8 × 10^−5^); *p* = 0.782
		Age	−1.3 × 10^−8^ (−6.6 × 10^−8^, 4.1 × 10^−8^); *p* = 0.647	6.0 × 10^−9^ (−1.3 × 10^−7^, 1.4 × 10^−7^); *p* = 0.929	1.8 × 10^−8^ (−1.6 × 10^−7^, 2.0 × 10^−7^); *p* = 0.842	2.3 × 10^−7^ (−1.7 × 10^−6^, 2.2 × 10^−6^); *p* = 0.817
		Relative VO_2max_	−6.7 × 10^−9^ (−3.9 × 10^−8^, 2.6 × 10^−8^); *p* = 0.681	1.8 × 10^−8^ (−6.5 × 10^−8^, 1.0 × 10^−7^); *p* = 0.666	2.5 × 10^−8^ (−8.4 × 10^−8^, 1.3 × 10^−7^); *p* = 0.646	7.4 × 10^−8^ (−1.1 × 10^−6^, 1.2 × 10^−6^); *p* = 0.899
		Mental Health	1.3 × 10^−7^ (−5.2 × 10^−7^, 7.8 × 10^−7^); *p* = 0.696	2.3 × 10^−9^ (−1.6 × 10^−6^, 1.6 × 10^−6^); *p* = 0.998	−1.3 × 10^−7^ (−2.3 × 10^−6^, 2.0 × 10^−6^); *p* = 0.906	−9.4 × 10^−6^ (−3.2 × 10^−5^, 1.3 × 10^−5^); *p* = 0.410
	Occipital HbR	Sex (F–Ref)	−1.3 × 10^−7^ (−6.3 × 10^−7^, 3.7 × 10^−7^); *p* = 0.603	5.0 × 10^−8^ (−8.1 × 10^−7^, 9.1 × 10^−7^); *p* = 0.907	4.2 × 10^−7^ (−6.9 × 10^−7^, 1.5 × 10^−6^); *p* = 0.455	1.4 × 10^−5^ (−1.9 × 10^−6^, 3.0 × 10^−5^); *p* = 0.084
		Conc Hx (No–Ref)	−1.9 × 10^−8^ (−4.4 × 10^−7^, 4.0 × 10^−7^); *p* = 0.928	−4.4 × 10^−7^ (−1.2 × 10^−6^, 3.0 × 10^−7^); *p* = 0.240	−3.9 × 10^−7^ (−1.4 × 10^−6^, 5.8 × 10^−7^); *p* = 0.423	1.1 × 10^−6^ (−1.3 × 10^−5^, 1.5 × 10^−5^); *p* = 0.876
		Age	−1.0 × 10^−8^ (−4.8 × 10^−8^, 2.8 × 10^−8^); *p* = 0.604	−3.1 × 10^−8^ (−9.7 × 10^−8^, 3.6 × 10^−8^); *p* = 0.364	−1.1 × 10^−8^ (−9.8 × 10^−8^, 7.5 × 10^−8^); *p* = 0.796	2.7 × 10^−7^ (−9.4 × 10^−7^, 1.5 × 10^−6^); *p* = 0.657
		Relative VO_2max_	−1.8 × 10^−8^ (−4.2 × 10^−8^, 5.3 × 10^−9^); *p* = 0.127	5.3 × 10^−9^ (−3.6 × 10^−8^, 4.7 × 10^−8^); *p* = 0.799	−1.1 × 10^−8^ (−6.4 × 10^−8^, 4.3 × 10^−8^); *p* = 0.691	−6.0 × 10^−7^ (−1.4 × 10^−6^, 1.5 × 10^−7^); *p* = 0.115
		Mental Health	−7.1 × 10^−8^ (−5.2 × 10^−7^, 3.8 × 10^−7^); *p* = 0.756	−1.3 × 10^−7^ (−9.2 × 10^−7^, 6.6 × 10^−7^); *p* = 0.741	−1.5 × 10^−7^ (−1.2 × 10^−6^, 8.7 × 10^−7^); *p* = 0.771	−7.3 × 10^−6^ (−2.2 × 10^−5^, 7.2 × 10^−6^); *p* = 0.321
	Occipital THb	Sex (F–Ref)	−2.7 × 10^−8^ (−8.8 × 10^−7^, 8.3 × 10^−7^); *p* = 0.951	−2.6 × 10^−7^ (−2.3 × 10^−6^, 1.8 × 10^−6^); *p* = 0.800	−3.0 × 10^−7^ (−3.2 × 10^−6^, 2.6 × 10^−6^); *p* = 0.842	1.5 × 10^−6^ (−3.0 × 10^−5^, 3.3 × 10^−5^); *p* = 0.926
		Conc Hx (No–Ref)	9.1 × 10^−8^ (−6.7 × 10^−7^, 8.5 × 10^−7^); *p* = 0.813	−8.0 × 10^−7^ (−2.6 × 10^−6^, 1.0 × 10^−6^); *p* = 0.388	−8.5 × 10^−7^ (−3.4 × 10^−6^, 1.7 × 10^−6^); *p* = 0.505	−3.3 × 10^−6^ (−3.1 × 10^−5^, 2.4 × 10^−5^); *p* = 0.812
		Age	−2.3 × 10^−8^ (−9.1 × 10^−8^, 4.4 × 10^−8^); *p* = 0.498	1.9 × 10^−8^ (−1.4 × 10^−7^, 1.8 × 10^−7^); *p* = 0.815	4.1 × 10^−8^ (−1.9 × 10^−7^, 2.7 × 10^−7^); *p* = 0.724	2.6 × 10^−7^ (−2.2 × 10^−6^, 2.7 × 10^−6^); *p* = 0.834
		Relative VO_2max_	−1.8 × 10^−8^ (−5.9 × 10^−8^, 2.3 × 10^−8^); *p* = 0.395	1.6 × 10^−8^ (−8.3 × 10^−8^, 1.1 × 10^−7^); *p* = 0.754	3.1 × 10^−8^ (−1.1 × 10^−7^, 1.7 × 10^−7^); *p* = 0.666	4.7 × 10^−7^ (−1.0 × 10^−6^, 2.0 × 10^−6^); *p* = 0.537
		Mental Health	1.0 × 10^−7^ (−6.9 × 10^−7^, 9.0 × 10^−7^); *p* = 0.796	−1.2 × 10^−7^ (−2.1 × 10^−6^, 1.8 × 10^−6^); *p* = 0.901	−3.0 × 10^−7^ (−2.9 × 10^−6^, 2.3 × 10^−6^); *p* = 0.823	−1.4 × 10^−5^ (−4.3 × 10^−5^, 1.5 × 10^−5^); *p* = 0.350
Finger Tapping	Motor HbO	Sex (F–Ref)	−1.7 × 10^−8^ (−2.6 × 10^−7^, 2.2 × 10^−7^); *p* = 0.889	−3.0 × 10^−8^ (−3.1 × 10^−7^, 2.5 × 10^−7^); *p* = 0.833	1.8 × 10^−8^ (−4.3 × 10^−7^, 4.7 × 10^−7^); *p* = 0.937	1.1 × 10^−6^ (−1.7 × 10^−6^, 3.9 × 10^−6^); *p* = 0.441
		Conc Hx (No–Ref)	1.2 × 10^−7^ (−8.9 × 10^−8^, 3.3 × 10^−7^); *p* = 0.255	5.2 × 10^−8^ (−1.9 × 10^−7^, 3.0 × 10^−7^); *p* = 0.672	−4.7 × 10^−8^ (−4.5 × 10^−7^, 3.6 × 10^−7^); *p* = 0.819	−7.3 × 10^−7^ (−3.2 × 10^−6^, 1.7 × 10^−6^); *p* = 0.554
		Age	1.2 × 10^−8^ (−6.5 × 10^−9^, 3.1 × 10^−8^); *p* = 0.196	−8.0 × 10^−9^ (−3.0 × 10^−8^, 1.4 × 10^−8^); *p* = 0.470	−1.9 × 10^−8^ (−5.4 × 10^−8^, 1.7 × 10^−8^); *p* = 0.300	−6.2 × 10^−8^ (−2.8 × 10^−7^, 1.6 × 10^−7^); *p* = 0.577
		Relative VO_2max_	−6.4 × 10^−9^ (−1.8 × 10^−8^, 5.3 × 10^−9^); *p* = 0.281	−6.4 × 10^−9^ (−2.0 × 10^−8^, 7.0 × 10^−9^); *p* = 0.346	−7.5 × 10^−9^ (−2.9 × 10^−8^, 1.4 × 10^−8^); *p* = 0.492	−4.1 × 10^−8^ (−1.8 × 10^−7^, 9.2 × 10^−8^); *p* = 0.541
		Mental Health	2.1 × 10^−8^ (−2.0 × 10^−7^, 2.4 × 10^−7^); *p* = 0.854	3.2 × 10^−8^ (−2.3 × 10^−7^, 2.9 × 10^−7^); *p* = 0.806	−2.1 × 10^−8^ (−4.5 × 10^−7^, 4.1 × 10^−7^); *p* = 0.925	−3.1 × 10^−7^ (−2.9 × 10^−6^, 2.3 × 10^−6^); *p* = 0.812
	Motor HbR	Sex (F–Ref)	**3.2 × 10^−7^ (2.9 × 10^−9^, 6.5 × 10^−7^); *p* = 0.048**	3.3 × 10^−7^ (−6.8 × 10^−8^, 7.4 × 10^−7^); *p* = 0.102	−2.3 × 10^−9^ (−9.8 × 10^−8^, 9.4 × 10^−8^); *p* = 0.962	−2.0 × 10^−6^ (−4.2 × 10^−6^, 1.7 × 10^−7^); *p* = 0.070
		Conc Hx (No–Ref)	−1.1 × 10^−7^ (−4.0 × 10^−7^, 1.8 × 10^−7^); *p* = 0.451	−1.6 × 10^−7^ (−5.0 × 10^−7^, 1.9 × 10^−7^); *p* = 0.364	−5.2 × 10^−8^ (−1.4 × 10^−7^, 3.2 × 10^−8^); *p* = 0.220	−1.1 × 10^−7^ (−2.0 × 10^−6^, 1.8 × 10^−6^); *p* = 0.911
		Age	−1.2 × 10^−8^ (−3.7 × 10^−8^, 1.4 × 10^−8^); *p* = 0.369	−1.6 × 10^−8^ (−4.7 × 10^−8^, 1.5 × 10^−8^); *p* = 0.323	−4.3 × 10^−9^ (−1.2 × 10^−8^, 3.1 × 10^−9^); *p* = 0.250	6.1 × 10^−8^ (−1.1 × 10^−7^, 2.3 × 10^−7^); *p* = 0.489
		Relative VO_2max_	−5.9 × 10^−9^ (−2.1 × 10^−8^, 9.2 × 10^−9^); *p* = 0.440	−4.7 × 10^−9^ (−2.4 × 10^−8^, 1.4 × 10^−8^); *p* = 0.623	1.6 × 10^−9^ (−2.9 × 10^−9^, 6.2 × 10^−9^); *p* = 0.485	7.7 × 10^−8^ (−2.8 × 10^−8^, 1.8 × 10^−7^); *p* = 0.149
		Mental Health	−5.7 × 10^−8^ (−3.6 × 10^−7^, 2.5 × 10^−7^); *p* = 0.712	−1.2 × 10^−9^ (−3.6 × 10^−7^, 3.6 × 10^−7^); *p* = 0.995	4.9 × 10^−8^ (−4.1 × 10^−8^, 1.4 × 10^−7^); *p* = 0.283	−3.9 × 10^−8^ (−2.1 × 10^−6^, 2.0 × 10^−6^); *p* = 0.970
	Motor THb	Sex (F–Ref)	**2.9 × 10^−7^ (4.6 × 10^−8^, 5.4 × 10^−7^); *p* = 0.021**	1.2 × 10^−7^ (−2.2 × 10^−7^, 4.6 × 10^−7^); *p* = 0.478	−1.5 × 10^−7^ (−5.0 × 10^−7^, 1.9 × 10^−7^); *p* = 0.374	−1.0 × 10^−6^ (−3.3 × 10^−6^, 1.2 × 10^−6^); *p* = 0.366
		Conc Hx (No–Ref)	−1.3 × 10^−9^ (−2.2 × 10^−7^, 2.1 × 10^−7^); *p* = 0.990	−1.2 × 10^−7^ (−4.1 × 10^−7^, 1.7 × 10^−7^); *p* = 0.418	−1.3 × 10^−7^ (−4.3 × 10^−7^, 1.7 × 10^−7^); *p* = 0.399	−7.8 × 10^−7^ (−2.8 × 10^−6^, 1.2 × 10^−6^); *p* = 0.435
		Age	3.4 × 10^−10^ (−1.9 × 10^−8^, 2.0 × 10^−8^); *p* = 0.973	−1.5 × 10^−8^ (−4.1 × 10^−8^, 1.1 × 10^−8^); *p* = 0.268	−1.4 × 10^−8^ (−4.1 × 10^−8^, 1.3 × 10^−8^); *p* = 0.318	−3.3 × 10^−9^ (−1.8 × 10^−7^, 1.7 × 10^−7^); *p* = 0.971
		Relative VO_2max_	−9.3 × 10^−9^ (−2.1 × 10^−8^, 2.5 × 10^−9^); *p* = 0.121	−2.0 × 10^−9^ (−1.8 × 10^−8^, 1.4 × 10^−8^); *p* = 0.806	4.7 × 10^−9^ (−1.2 × 10^−8^, 2.1 × 10^−8^); *p* = 0.570	3.8 × 10^−8^ (−7.1 × 10^−8^, 1.5 × 10^−7^); *p* = 0.493
		Mental Health	−1.5 × 10^−8^ (−2.4 × 10^−7^, 2.1 × 10^−7^); *p* = 0.896	−5.9 × 10^−8^ (−3.7 × 10^−7^, 2.5 × 10^−7^); *p* = 0.708	−2.7 × 10^−8^ (−3.5 × 10^−7^, 3.0 × 10^−7^); *p* = 0.868	−3.9 × 10^−7^ (−2.5 × 10^−6^, 1.7 × 10^−6^); *p* = 0.710

Significant values are displayed in bold font. Area under the curve (AUC), oxygenated hemoglobin (HbO), deoxygenated hemoglobin (HbR), total hemoglobin (THb), concussion history (Conc Hx), positive (+), negative (−), maximal oxygen update (VO_2max_).

**Table 4:** Electroencephalography frequency band power linear mixed effect models coefficients and 95% confidence intervals in 113 participants (77% female) during visual- and motor-based neurovascular coupling tasks.

Task	Band	Variable	Baseline	Peak	Absolute Increase	Relative Increase	AUC
Waldo	Alpha	Sex (F–Ref)	1.9 (−11.9, 15.7); *p* = 0.784	12.6 (−51.8, 77.0); *p* = 0.698	11.4 (−83.9, 106.7); *p* = 0.813	9.3 (−42.0, 60.7); *p* = 0.720	−37.0 (−98.7, 24.7); *p* = 0.237
	(8–12 Hz)	Conc Hx (No–Ref)	2.6 (−9.6, 14.9); *p* = 0.670	10.3 (−47.0, 67.6); *p* = 0.723	−43.6 (−126.5, 39.3); *p* = 0.299	8.3 (−37.2, 53.8); *p* = 0.718	−30.9 (−86.6, 24.8); *p* = 0.273
		Age	0.8 (−0.3, 1.9); *p* = 0.149	2.7 (−2.4, 7.8); *p* = 0.289	−3.1 (−10.5, 4.3); *p* = 0.409	1.9 (−2.2, 5.9); *p* = 0.356	4.3 (−0.7, 9.2); *p* = 0.091
		Relative VO_2max_	0.2 (−0.5, 0.9); *p* = 0.603	0.3 (−2.7, 3.4); *p* = 0.824	−3.0 (−7.6, 1.5); *p* = 0.190	0.4 (−2.2, 3.0); *p* = 0.767	0.2 (−2.8, 3.3); *p* = 0.893
		Mental Health	−3.6 (−16.4, 9.2); *p* = 0.574	−19.2 (−80.1, 41.8); *p* = 0.534	−20.1 (−107.0, 66.9); *p* = 0.648	−15.9 (−63.1, 31.3); *p* = 0.505	−17.7 (−76.8, 41.4); *p* = 0.554
	Low Beta	Sex (F–Ref)	−0.4 (−10.6, 9.7); *p* = 0.937	25.1 (−68.5, 118.7); *p* = 0.596	20.4 (−69.3, 110.0); *p* = 0.653	26.5 (−58.8, 111.9); *p* = 0.539	−27.8 (−84.7, 29.1); *p* = 0.335
	(12–16 Hz)	Conc Hx (No–Ref)	0.3 (−8.9, 9.4); *p* = 0.950	30.3 (−53.0, 113.6); *p* = 0.472	15.0 (−64.7, 94.7); *p* = 0.710	27.8 (−48.1, 103.8); *p* = 0.469	−14.8 (−65.9, 36.2); *p* = 0.566
		Age	0.7 (−0.1, 1.6); *p* = 0.072	3.4 (−4.0, 10.8); *p* = 0.367	−0.5 (−7.5, 6.5); *p* = 0.889	2.6 (−4.2, 9.4); *p* = 0.455	**5.6 (1.0, 10.1); *p* = 0.017**
		Relative VO_2max_	0.2 (−0.3, 0.6); *p* = 0.535	1.8 (−2.7, 6.3); *p* = 0.429	0.6 (−3.6, 4.9); *p* = 0.760	1.4 (−2.7, 5.6); *p* = 0.493	1.4 (−1.4, 4.1); *p* = 0.330
		Mental Health	−3.0 (−12.6, 6.6); *p* = 0.532	−32.2 (−120.4, 56.0); *p* = 0.471	−15.4 (−99.5, 68.7); *p* = 0.717	−31.6 (−111.1, 47.9); *p* = 0.432	−25.6 (−79.5, 28.3); *p* = 0.348
Finger Tapping	Alpha	Sex (F–Ref)	0.3 (−7.4, 7.9); *p* = 0.948	24.6 (−23.0, 72.2); *p* = 0.308	11.0 (−188.9, 210.9); *p* = 0.913	24.9 (−16.6, 66.3); *p* = 0.237	35.6 (−108.1, 179.3); *p* = 0.624
	(8–12 Hz)	Conc Hx (No–Ref)	1.4 (−5.4, 8.2); *p* = 0.686	21.7 (−20.5, 63.9); *p* = 0.310	−75.4 (−253.8, 103.1); *p* = 0.404	20.0 (−17.1, 57.2); *p* = 0.287	35.8 (−89.9, 161.4); *p* = 0.574
		Age	−0.3 (−0.9, 0.3); *p* = 0.372	−0.9 (−4.7, 2.8); *p* = 0.620	−6.9 (−22.7, 8.9); *p* = 0.386	−0.7 (−4.0, 2.6); *p* = 0.675	4.4 (−7.0, 15.7); *p* = 0.447
		Relative VO_2max_	−0.0 (−0.4, 0.4); *p* = 0.986	0.6 (−1.8, 2.9); *p* = 0.643	−3.0 (−12.3, 6.2); *p* = 0.518	0.5 (−1.5, 2.5); *p* = 0.601	5.2 (-1.8, 12.2); *p* = 0.146
		Mental Health	−0.4 (−7.6, 6.8); *p* = 0.910	−12.4 (−57.3, 32.5); *p* = 0.585	−2.4 (−195.2, 190.3); *p* = 0.980	−13.4 (−52.2, 25.3); *p* = 0.494	−40.3 (−171.2, 90.6); *p* = 0.543
	Low Beta	Sex (F–Ref)	−1.4 (−6.8, 4.1); *p* = 0.623	14.6 (−25.6, 54.8); *p* = 0.472	80.6 (−65.8, 226.9); *p* = 0.277	16.3 (−19.5, 52.1); *p* = 0.370	2.7 (−93.8, 99.3); *p* = 0.955
	(12–16 Hz)	Conc Hx (No–Ref)	−0.6 (−5.5, 4.3); *p* = 0.802	12.5 (−23.6, 48.6); *p* = 0.493	3.3 (−128.2, 134.7); *p* = 0.961	13.4 (−18.9, 45.7); *p* = 0.412	−10.3 (−96.8, 76.1); *p* = 0.813
		Age	−0.1 (−0.5, 0.4); *p* = 0.774	−0.3 (−3.5, 2.9); *p* = 0.861	−3.5 (−15.2, 8.1); *p* = 0.552	−0.2 (−3.1, 2.6); *p* = 0.877	5.1 (−2.6, 12.7); *p* = 0.191
		Relative VO_2max_	−0.0 (−0.3, 0.2); *p* = 0.875	0.2 (−1.8, 2.3); *p* = 0.831	−2.2 (−8.9, 4.5); *p* = 0.519	0.2 (−1.5, 1.9); *p* = 0.827	2.2 (−2.4, 6.7); *p* = 0.344
		Mental Health	−0.7 (−5.9, 4.5); *p* = 0.797	−11.5 (−49.7, 26.7); *p* = 0.551	−47.3 (−186.2, 91.6); *p* = 0.501	−11.1 (−45.1, 22.8); *p* = 0.517	−16.3 (−106.6, 74.0); *p* = 0.722

Significant values are displayed in bold font. Area under the curve (AUC), concussion history (Conc Hx), positive (+), negative (−), maximal oxygen update (VO_2max_).

## Data Availability

Data is available upon reasonable request to the authors.

## Abbreviations

The following abbreviations are used in this manuscript:

EEG	Electroencephalography
fNIRS	Functional Near-Infrared Spectroscopy
TCD	Transcranial Doppler
AUC	Area Under the Curve
MCAv	Middle Cerebral Artery velocity
PCAv	Posterior Cerebral Artery velocity
CBv	Cerebral Blood Velocity
CO_2_	Carbon Dioxide
P_ET_CO_2_	Partial Pressure End-Tidal Carbon Dioxide
HbO	Oxygenated Hemoglobin
HbR	Deoxygenated Hemoglobin
THb	Total Hemoglobin
VO_2max_	Maximal Oxygen Uptake
